# “Everything Changed, Would You Like Me to Elaborate?”: A Qualitative Examination of the Impact of the COVID-19 Pandemic on Community Participation Among Young Adults with Early Psychosis and Their Families

**DOI:** 10.1007/s10597-022-01049-y

**Published:** 2022-12-02

**Authors:** Sapana R. Patel, Ana Stefancic, Iruma Bello, Shannon Pagdon, Elaina Montague, Melody Riefer, Jamaitreya Lyn, Joan Archard, Reanne Rahim, Leopoldo J. Cabassa, Chacku M. Mathai, Lisa B. Dixon

**Affiliations:** 1 The New York State Psychiatric Institute, New York, NY 10032 USA; 2 Department of Psychiatry, Columbia University Vagelos College of Physicians and Surgeons, New York, NY 10032 USA; 3 Center for Psychiatric Rehabilitation, Boston University, Boston, MA 02215 USA; 4 Brown School of Social Work, Washington University in St. Louis, St. Louis, MO 63130 USA

**Keywords:** Community, Participation, COVID-19, Young adults, Early, Psychosis

## Abstract

OnTrackNY provides early intervention services to young people with early psychosis throughout New York State. This report describes the impact of the COVID-19 pandemic on community participation of OnTrackNY program participants and their families. Thirteen participants and nine family members participated in five focus groups and three individual semi-structured interviews. Data were analyzed using a summary template and matrix analysis approach. Major themes highlight the negative impacts of the pandemic with reports of decreased socializing or using online means to connect, unemployment, challenges with online learning and a decrease in civic engagement. Positive impacts include more time to deepen connections with family and valued friendships and engage in activities that promote wellness and goal attainment. Implications for coordinated specialty care programs include adapting services to promote mainstream community integration and creating new strategies for community involvement of young people within a new context brought forth by the pandemic.

The International Classification of Functioning, Disability and Health framework defines community participation as the involvement in social activities including work, education, community, social and civic life (e.g., wellness, recreation, leisure activities and faith-based activities) and interpersonal interactions and relationships (WHO, 2001). Community participation in social, recreational and occupational activities is vital during young adulthood (Martel & Fuchs, 2017), especially for young adults with serious mental illness (SMI) who commonly experience loss of community participation due to isolation and social withdrawal (Addington & Addington, 2008) after onset of illness. Young adults with SMI are also at greater risk for health problems and poorer functional outcomes (Kaplan et al., 2012).

Community participation fosters social support and critically important recovery skills including self-determination, personal agency and choice shown to be associated with positive physical, cognitive, and mental health and quality of life outcomes among individuals with SMI (Burns-Lynch et al., 2016; Kaplan et al., 2012; Salzer & Baron, 2016). Advocates and researchers have emphasized that mental health programs that serve young adults with early psychosis, such as coordinated specialty care (CSC), integrate services that support community participation (Sale et al., 2018; Thomas et al., 2020). In the United States, CSC is an evidence-based multi-element team-based approach shown to reduce relapse and improve outcomes for youth and young adults with early psychosis (Dixon et al., 2015; Kane et al., 2016). In a recent modified e-Delphi study by Thomas and colleagues (2022), CSC experts identified practices at the service provider, practice and organizational-levels that promote community participation. For CSC programs, this includes routinely meeting with participants in community-based settings, having detailed knowledge about community resources and participation options in each participation area, working to reduce structural barriers to community participation, promoting the development and use of natural supports of peers and family members, and having CSC program statements that explicitly promote community participation. They also highlighted the importance of educating clinical staff about the positive outcomes associated with community participation.

OnTrackNY is a nationally recognized model of CSC that has been implemented across New York state (Bello et al., 2017). OnTrackNY offers medication management, care management, cognitive-behaviorally oriented therapy for psychosis and other comorbidities, and evidence-based interventions to support community participation including family psychoeducation and support, supported employment and education, and peer support services (Bello et al., 2017; Heinssen et al.,^ 2014^). OnTrackNY teams use a shared decision making approach to work with participants in the clinic, their homes, and in their communities to support participation in mainstream, developmentally appropriate activities that facilitate the attainment of work, school and life goals. Individuals who receive OnTrackNY services have shown improved rates of social and occupational functioning, demonstrate significant increases in education and employment attainment, and experience a decrease in hospitalization rates (Nossel et al., 2018). Given the success of CSC programs, federal investments have supported the coordination of CSC programs nationwide to create a learning healthcare system that achieves quality, safety and value in early intervention services for young adults with early psychosis called the Early Psychosis Intervention Network (EPINET) (Humensky et al., 2020). OnTrackNY is a scientific hub of EPINET.

Little is known about the impact of COVID-19 on community participation among young adults with early psychosis. The existing literature on the impact of the pandemic has almost entirely focused on symptom exacerbation, relapse rates, and experiences with telehealth (Chaudhry et al., 2021; LeComte et al., 2021; Meyer-Kalos et al., 2020; Pires de Oliveira et al., 2021; Szmulewicz et al., 2021). One study that used thematic analysis of Reddit posts to understand the experiences of people with psychosis during the pandemic revealed barriers to community participation including a lack of opportunities to participate in wellness activities in the community (e.g., gym, martial arts classes) and changes in social relationships during the pandemic (e.g., break-ups with significant others, increased isolation at home) (Lyons et al., 2021). A greater understanding of how the COVID-19 pandemic impacted community participation among young adults with early psychosis and how CSC programs can support community participation can inform care systems about how to mitigate disruption and harness recovery supports for this vulnerable population.

Informed by the principles of community-based participatory research (CBPR) (Minkler & Wallerstein, 2008), we partnered with graduates of the OnTrackNY program and a family member to conduct a quality improvement (QI) project to learn about the impact of the COVID-19 pandemic on community participation among CSC program participants. We sought to understand how the pandemic impacted different aspects of community participation and how OnTrackNY supported community participation goals of young people during the pandemic.

## Methods

### Amplify OnTrackNY

Amplify OnTrackNY is the stakeholder engagement program of EPINET OnTrackNY. Its mission is to engage and partner with stakeholders (e.g., OnTrackNY participants, family members, providers, trainers, policy makers) to create knowledge and facilitate change that will improve care delivery and outcomes for young adults with early psychosis (Humensky et al., 2020). The Amplify team includes individuals with early psychosis lived experience, those with expertise in research and evaluation (e.g., qualitative and implementation science researchers, project manager), and leadership at OnTrack Central, an intermediary organization that provides training and implementation support to OnTrackNY teams. Amplify OnTrackNY leverages existing OnTrackNY councils, including the Youth and Young Adult Leadership Council (YYLC), the Family Advisory Council (FAC), and Provider Council (PC), all of which serve as forums for stakeholders to provide feedback about the OnTrackNY program.

### Collaboration with OnTrackNY Graduates and Family Member

To further align this project with principles of inclusion, co-learning, and capacity-building (Minkler & Wallerstein, 2008), the Amplify team collaborated with two graduates of OnTrackNY and a family member of an OnTrackNY participant in all phases of the project, from conceptualization, recruitment and data collection to data interpretation and dissemination of findings. The graduates and family member were compensated for their time and supported by the project manager and a team member with lived experience through bi-weekly meetings focused on clarifying roles, co-planning participant focus groups, training in focus group facilitation and practicing focus group procedures.

### Sample

A convenience sampling approach was used for recruitment. Eligible participants were those who were English speaking and currently enrolled in OnTrackNY services or who had received OnTrackNY services during the pandemic but had since graduated. Eligible family members were those who were English speaking and had a relative (e.g., child, sibling) who was an OnTrackNY participant during the pandemic. Recruitment consisted of flyers and presentations by Amplify team members and the OnTrackNY graduates and family member at provider team meetings, participant/family groups, and council meetings (YYLC, FAC), as well as informing those who expressed interest in being contacted about Amplify activities. All interested individuals were informed of participation requirements and that the information they provide may be used for research purposes, and screened over the telephone to assess for eligibility. If eligible, they provided verbal consent for participation and audio recording. The project was conducted as part of quality improvement for EPINET OnTrackNY and was determined to be non-human subjects research by the New York State Psychiatric Institute Institutional Review Board.

### Data Collection

We chose focus groups as our primary method of data collection to (1) capture information about the impact of COVID-19 rapidly and inform program development and (2) since many individuals experienced challenges during this time, we surmised that hearing from other participants and families regarding their struggles might encourage sharing and promote discussion. However, to avoid excluding the perspectives of individuals whose schedules may not accommodate a focus group or who may have felt uncomfortable sharing in a group, the option of one-on-one interviews was also offered. Interview guides were the same for both modalities.

Three focus groups and two individual semi-structured interviews were conducted with OnTrackNY participants and graduates, while two focus groups and one interview were conducted with family members. Due to the pandemic, all qualitative data collection was conducted online via Zoom, a HIPAA-compliant video conference platform, with one interview completed by phone. With the exception of one family interview, all data collection was led by a project team member with lived experience (e.g., OnTrackNY graduates, family members, individuals with lived experience of psychosis). Each participant and family focus group consisted of three to four participants or families and was jointly facilitated by a team member with lived experience and one of the two OnTrackNY graduates or family member, with the project manager providing administrative support. One semi-structured interview with the project manager was conducted for a family member who could not attend the scheduled focus group. Data collection occurred between June 2021 and March 2022.

Interview and focus group guides were developed collaboratively by the OnTrackNY graduates, family member and the Amplify team. The guides explored how OnTrackNY participants’ lives had changed during the pandemic, including in areas such as personal relationships and social support, involvement in work and school, and use of free time during the pandemic. Sample questions included: “What has your participation in work or school been like during the pandemic?” and “How would you describe your relationships with family, friends, and loved ones throughout the pandemic?” The family focus group/interview guide explored how the pandemic impacted the families and the support they needed as well as their experiences with OnTrackNY during the pandemic and suggestions for program improvement. Sample questions included: “How did the pandemic impact your life and that of your loved one (for example, their participation in work or school)?” “In what ways did your loved one need support during the pandemic? and “Did the support that your loved one needed from the team change at all during the pandemic. If yes, how so?” Focus groups and interviews were audio-recorded, professionally transcribed verbatim, de-identified, and reviewed for accuracy by the project manager. Participants and family members also completed a voluntary web-based survey via Qualtrics to self-report demographic characteristics.

### Data Analysis

**Fig. 1:**
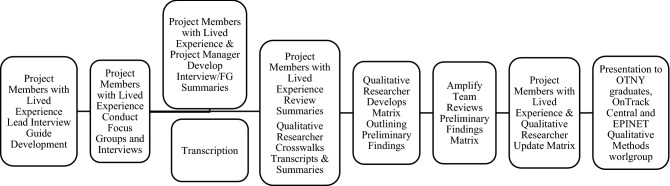
Collaborative and pragmatic rapid qualitative research

Descriptive statistics were used to summarize available demographic information. Interview and focus group data were analyzed using a summary template and matrix analysis approach to categorize participant and family members’ responses along key topics (Fig. 1). This analytical approach is a rigorous but pragmatic method for rapidly extracting and reducing qualitative data, allowing systematic synthesis and cataloging of content into a template of key topics (Abraham & Van Tiem, 2021; Averill, 2002; Gale et al., 2019). First, draft summaries of participant and family focus groups/interviews were developed by a team member with lived experience and project manager, respectively. This involved summarizing the content discussed along key interview topics (e.g., impact on social relationships, school/work participation). Second, these summaries were reviewed by the OnTrackNY graduates and family member and team member with lived experience. To further ensure that relevant information from interviews and focus groups was captured in the summaries, a qualitative researcher reviewed each transcript and revised summaries as needed. Third, the qualitative researcher developed a draft table template to document content emerging from each focus group/interview. Matrix column headings were chosen to facilitate rapid extraction of transcript content by life domain and represented key topics of the interview guide, with some additional category breakdowns (e.g., social connections: friends/peers, family; work/school and other wellness or recreational activities). The qualitative researcher systematically extracted information from each summary and entered it into the draft matrix using short telegraphic phrases (i.e., charting) (e.g., family: together more, got closer; work: lost job, looking for work). The draft matrix was then evaluated by the Amplify team and subsequently updated by a team member with lived experience and qualitative researcher based on a final review of transcripts. Finally, multiple Amplify team members reviewed and discussed the completed matrix to identify patterns and contrasts both within and across life domains and participants, with the OnTrackNY graduates reviewing a preliminary presentation of results.

Strategies for maximizing the rigor of this pragmatic approach included progressively reducing the data using a series of defined steps (e.g., transcribing, summarizing, charting); using multiple team members at each step to extract, reduce, categorize, and interpret the data; conducting frequent debriefing meetings throughout data collection and analysis; and keeping an audit trail (Creswell & Creswell, 2017; Padgett, 2012). Trustworthiness of our results was verified using member checking activities (Birt et al., 2016), wherein the team member with lived experience and OnTrackNY graduates presented findings from the participant qualitative data at a YYLC meeting open to all OnTrackNY participants or graduates, at an OnTrack Central meeting and to the EPINET Qualitative Research Methods workgroup to solicit their feedback. Feedback was generally positive and focused on suggestions to expand on understanding strategies for CSC participant engagement and community participation using a combination of in-person and online approaches.

## Results

### Sample Characteristics

Thirteen OnTrackNY participants or graduates and nine family members completed focus groups or interviews. Sample characteristics can be found in Table 1. Themes and subthemes describing the impact of the pandemic clustered around five main domains of the International Classification of Functioning, Disability and Health framework (WHO, 2001) including family, social connections, education, work and wellness and recreation and are described below. A sample matrix can be found in Table 2.

**Table 1:** OnTrackNY Participant and Family Member Characteristics

	OnTrackNY participants (n = 13) n (%)^a^	OnTrackNY Family members (n = 9) n (%)^a^
Age group		
18–24	8 (61.5)	
25–34	4 (30.8)	1 (11.1)
35–44		1 (11.1)
45–54		2 (22.2)
55–64		1 (11.1)
≥ 65		1 (11.1)
Missing	1 (7.7)	3 (33.3)
Gender		
Female	5 (38.5)	7 (77.8)
Male	5 (38.5)	
Prefer to not say/Missing	3 (23.1)	2 (22.2)
Race/Ethnicity		
Asian (non-Hispanic)	3 (23.1)	2 (22.2)
Black (non-Hispanic)	2 (15.4)	3 (33.3)
Hispanic	3 (23.1)	
White (non-Hispanic)	3 (23.1)	2 (22.2)
Prefer to not say/Missing	2 (15.4)	2 (22.2)
Degree or certification		
High school diploma/GED	2 (15.4)	1 (11.1)
Some college	5 (38.5)	
College degree	4 (30.8)	2 (22.2)
Post-graduate degree	1 (7.7)	4 (44.4)
Missing	1 (7.7)	2 (22.2)
Geographic region		
New York City	12 (92.3)	6 (66.7)
Western New York		1 (11.1)
Missing	1 (7.7)	2 (22.2)
Months in OnTrackNY Program mean (SD)	13.5 (6.17)	27.0 (6.7)

^a^Percentages may not total 100% due to rounding

**Table 2:** Impact of COVID-19: Sample Analysis Matrix

Participant/family	Social connections: friends, peers	Social connections: family	Work/employment	School/education	Activities (including Health/Wellness)
Family Interview	Positives: OnTrackNY outings in later phases - social, activities—walks; Peer group virtually *“They loved [the outings]. It’s exciting for them, it’s more change, they get to participate with others, it becomes a social thing.“* Challenges: Generally, less interaction with others, not wanting to go out even in later phases *“They just changed during the COVID, it made them more lazy. Their interaction with other people changed. Before, they would go out, somebody would call, let’s go out. Now it’s like, nobody wants to call or go out, nobody wants to—they just wanna stay home.“*	Challenge: Unable to see grandfather as frequently as before COVID when used to see a few days a week More dependent on themselves and the parents now “The progress, it slowed it down. They weren’t able to go to school, and everything changed, and the change made them more dependent on themselves and they were more dependent on us, also.“	Not mentioned	Challenges: Was failing school with shift to remote, graduated but 6 months late Attributes to less accountability with remote school *“They wouldn’t go to class. It’s not easy…since you’re home you don’t wanna do it because nobody’s watching you. So, you just end up not doing it.“*	Challenges: Being locked in the house changed the person - became little lazy, low motivation to go out, hard to break habit of not going out *“You’re not used to being locked in the house. Once they made you stay in the house, it changed the person—it changed the attitudes, made them a little lazy. They didn’t want to go out afterwards when the situation had got better.“*
Participant FG	Positives: Able to determine who cares and who does not, figure out who to keep in contact with. Found it surprising that people close were supportive. Keeps relationship with old coworkers and classmates to help find job opportunities *“I figured out the people who—if I haven’t talked to anyone during this phase, I’m definitely never going to talk to them again. If I see them, I’ll make conversation but I kinda know the people who I care about and the people who care enough to respond to reach out to me.“*	Challenges: Dad works in hospital which was nerve-wracking. Positives: didn’t realize how much family cared about me till psychosis; how much I depend on them and how much they support me, care about me	Challenges: Turned down jobs - not in right mental space, boring job that puts me to sleep	Positives: doing step-by-step tutorials, it’s a different way of learning- in a good way *“I’m not doing college courses…tutorials but they kind of walk you step-by-step through it [online], and I really enjoy that; I think it’s a very different way of learning. And it’s how I wish I had done everything ever.“*	Challenges: Does not engage in physical activity as much, spends more time sleeping (to make up for lack of engagement) Trying to participate in healthier activities like reading, took ownership of space and redecorated *“it’s been a realization I just need to focus on myself and be a positive force, or person to the people around me, and just general health stuff, mental health, nutrition, sleep, mindfulness…that’s what I’m focused on.“*

### Family

Many participants and family members reported improved family relationships and feeling more connected due to increased time spent together and proximity to each other. One participant described:

We had the opportunity to spend even more time with family, and actually [have] that bond grow even further. And it allowed us to lean on each other a little bit more and support each other throughout this pandemic. And really be there for each other emotionally… have meals together and play games together. So, we’re definitely a lot closer than before.

Family members who were able to work from home highlighted the benefit of being physically present for their loved one and able to provide on-going encouragement or support, “It really helped him because we were able to be home. We were able to really interact with him and help him a lot.” One parent highlighted the importance of support from a sibling:

[Her brother] understood better than me what was going on...with the pandemic, and how she just needed to realize that she was loved and that she was not alone. This is not just your thing. This is our thing.

While this extra family support was generally considered positive, there was also a concern that no longer having in-person access to other types of social network supports meant that participants were “more dependent on us [the parents].” For some participants, spending time with family and parents was challenging since it was harder for them to reach other supportive people that they wanted to connect to when they felt troubled at home. One sibling of a participant described the challenge of not knowing how to support her brother:

I feel like my brother is stuck...And like you don’t wanna push too hard either. So, just finding that loving nudge to get out there. And I think it’s hard. Especially as a family member...if OnTrack could help...[offer] direction on that.

### Social Relationships

Unlike the increased time spent in-person with family, with very few exceptions, participants’ interactions with friends, colleagues, and peers largely took place online or by phone. Many adapted to the challenges of not seeing others in person by playing online games with friends or just talking online:

Since I was spending so much time playing games online, I managed to make a lot of good friends, but we’ve basically been spending almost every day with each other now…playing…games online.

Participants shared that not being able to see peers and friends in a school environment made it more difficult to maintain social relationships and took extra effort. As a result, participants reported needing to “simplify who I really would like to spend my time and energy with.” Some reported that they “figured out” who their true friends were during this time, emphasizing the need to invest in those friendships with people who made effort to remain in touch and who showed that they cared:

I don’t have friends that much, but I try to keep those friends that are close to me as close as possible...but those friends that don’t even care about me, I leave them aside.

### Education

Participants described diverse experiences with focus and ability to engage in school and online learning. They described challenges such as feeling that the educational material was harder to learn without being in a classroom, experiencing a loss of focus and difficulty collaborating with peers online, and facing challenges with coursework that was not adjusted to account for diverse home learning environments. One participant explained:

All my classes within grad school are online now…they don’t realize everyone doesn’t have the same testing environment…and I think it’s been more difficult due to a lot of the concentration difficulties that come with psychosis with having to study from home.

In addition to the increased educational struggles and need for extra academic support during this time, one family member shared the challenges of advocating for her son to get appropriate academic support services:

And I can tell you it’s been a lot of work through me having to work with the college just to get him any kind of services. Even though we went through the disability services, even though our therapist and his doctor through OnTrack were able to send everything over there, they didn’t really provide him anything.

Challenges associated with online learning often led to lower grades or slowed academic progress, with some participants struggling for longer periods of time, while others were able to graduate after a period of adjustment: “It’ll be six months late, but [my child] graduated high school.” A few participants commented on the positive impacts of online learning on their attention and engagement. One participant noted more active learning with online courses and tutorials, “I think it’s a very different way of learning. And it’s how I wish I had done everything ever.”

### Work

Many participants who had been employed prior to the pandemic experienced job loss or furlough. They held jobs in fields such as real estate, catering, or childhood education, that were deemed non-essential per pandemic regulations at the time or jobs that could not easily transition to remote work: “I lost my job since we couldn’t go outside, my job wasn’t one of those essential worker things, I lost a source of income.” In addition to straining finances, participants emphasized how job loss impacted their motivation and ability to engage in other meaningful activity: “when I was doing real estate I was not allowed to work because I wasn’t essential. So, that’s why…I guess, [I] spiraled into doing basically nothing.”

Family members commented similarly on the impact of their loved one’s work goals being put on hold due to the pandemic, as exemplified by one family member:

He was furloughed…He lost focus. He lost anything to do with feeling confident and leaving the house and being pushed into a social setting…So, someone with schizophrenia, then you’re left with all your symptoms. You have virtually nothing but symptoms to deal with. So, that was really hard on him, and he was not able to go back.

Despite the challenges associated with job loss and furloughs, after a period of time, some ultimately returned to their jobs while others decided to advance their skills through online education and/or looking for new work:

I came to the realization that I wanted to be more productive and do something with my life…instead of going and get a job route…[I] sort of became a student again. [Now] I’m looking for jobs and it’s a little bit tough…it’s just something that I have to keep working on and keep going… [OnTrackNY supported me] emotionally during my stressful times…[they] gave me a lot of strength during my struggles.

### Wellness and Recreation

Participants reported having a lot of unstructured free time during early stages of the pandemic and mostly spent time streaming video content, playing video games, and sleeping.

Every waking hour was free time. Because I didn’t really have a goal. I was just trying to survive… like everyone else—I was binge-watching Netflix, playing video games with my [relative]. And just trying to get as much sleep as possible.

Acknowledging that they had become more sedentary, some participants commented on the frustrations of weight gain, “The biggest impact has been because I’ve gained a few pounds…obviously, the gyms no longer became an option,” and family members expressed concern:

I made a payment to the gym…when they were able to go, they didn’t feel like going…They didn’t wanna go out as much, at all. Not even to the store. I’d have to push them to go to the store or I would have to go with them.

While some participants continued to struggle with free time and inactivity, most described realizing that they needed to re-engage with goals and more meaningful activity: “I was just playing video games every day or watching YouTube. And originally, I thought that’s what I wanted, and after a few months of doing it, it was like oh, this kinda sucks…” One family member commented: “my son’s actually on his way out to Taekwondo. He started a Taekwondo class. Loves it…he plays basketball a couple times a week. He goes to Taekwondo and he’s taking a class. Those are wins.”

Participants made efforts to participate in wellness activities they could practice on their own when physical distancing was recommended, such as taking walks, doing yoga, learning new hobbies or skills. Many participants referenced this as learning to create a new “balance” in their lives:

I realized how much energy [playing video games and watching shows] really drained from me…my passion and purpose [then] really aligned with getting stronger each day, exercising each day…maintaining that energy to stretch, play ball, my favorite hobbies…even in this COVID state, I’ve been able to be balanced.

Finally, discussions about wellness and recreation highlighted the importance and potential for these activities to serve as a way to connect with others. For example, a family member describes the benefits of group community recreational activities within OnTrackNY and others to exercise with:

When the situation got a little better, they were taken on field trips, walks [by OnTrackNY]...[My children] loved it. It’s exciting for them, it’s more change, they get to participate with others, it becomes a social thing. They still have it now...It gets them out.

Another example was a health-related blog that enhanced a participant’s social support:

I’d say my social support has grown stronger…I created a page—I feel like it was a little before the pandemic—but I’ve been posting on [my blog] off and on throughout the year just to give general health updates about myself…so I’ve been more open about my experiences.

## Discussion

This study fills a critical knowledge gap about the impact of the COVID-19 pandemic on community participation among CSC participants and their family members. Participants and family members described positive and negative impacts on all domains of community participation (WHO, 2001). Negative impacts include decreased socializing or using online means to connect with friends, unemployment, challenges with online learning and a decrease in civic engagement. Positive impacts include more time to deepen connections with family and valued friendships and engage in new activities that promote wellness and goal attainment. We also learned about how CSC program participants, families and providers supported community participation during a time of crisis and community shutdown during the pandemic.

Young adults with early psychosis in this sample experienced similar challenges and negative impacts due to pandemic restrictions (e.g., job loss concerns, isolation, lack of motivation) compared to other studies with samples of young adults in general (Ammar et al., 2020; Birmingham et al., 2021; Molock & Parchem, 2021) except for challenges with online learning which may be due to impairments in cognitive functioning related to psychosis and families’ involvement in advocating for educational accommodations with schools.

Findings from this qualitative examination have several implications for how CSC services can support community participation. Based on feedback from OnTrack Central, we provide examples of how OnTrackNY teams supported community participation. While family relationships were generally positive, descriptions of greater family presence and involvement across multiple domains of participants’ lives could nevertheless increase caregiver burden. To address this, we learned that teams started additional online family support groups which increased family engagement and provided families skills in problem-solving they could use to help their loved ones continue to pursue their goals.

Educational and employment pursuits are a cornerstone of community participation and a primary target of CSC interventions, yet they suffered the most during the pandemic. To support participants’ employment goals, OnTrackNY Supported Employment and Education Specialists (SEES) engaged in online job development, assembled a repository of information of online resources for employment, and supported individuals to complete job applications online and prepare for online job interviews. To support participants’ education goals, OnTrackNY SEES and clinicians helped young people integrate cognitive intervention-based compensatory strategies to facilitate engaging in online classes, including addressing issues of organization, attention and navigating the online environment.

Participants and families highlighted the need to address inactivity and social isolation. In a virtual environment, OnTrackNY peer specialists promoted civic engagement by sharing opportunities for online participation in civic groups; online forums for connecting and experiencing art, music and culture; social networking; and virtual faith-based or spiritual groups. OnTrackNY teams also offered online socialization groups to allow participants an opportunity to connect with others. Using online means for greater involvement in digital participation, whether the activities are intended for spending time alone or for interacting socially with others, may also empower people during their recovery (Shpigelman et al., 2021).

Almost all participants experienced an initial period of retreat from activities and stagnation in multiple life domains during early phases of the pandemic. However, many subsequently reached a point where they used the pandemic as a time to reflect and reassess their lives and make positive changes in various life domains, including pursuing new education or career opportunities that they were passionate about, investing time and energy in relationships that were important to them, and pursuing wellness activities as positive coping mechanisms. Nevertheless, others continued to struggle, perceiving setbacks or minimal progress concerning their goals and life pursuits, and significantly limited participation with peers and the broader community. For these individuals, expanding opportunities for support from OnTrackNY Peer Specialists may be especially crucial as they developed strategies that fostered activity and social connections. Some other strategies may include using shared decision making to discuss the risks and benefits associated with engaging in community-based activities and connecting more with social support networks.

This project has several strengths. First, this qualitative study captured a range of community participation experiences among a racially and ethnically diverse sample of CSC participants, graduates and family members. Second, CSC graduates and a family member co-led this project from conceptualization to dissemination of study findings which increases the relevance of this project and provided an opportunity for participatory research which may further facilitate community participation (Chan et al., 2017; Jordan et al., 2018). There are also limitations to note, primarily due to the recruitment of a small geographically concentrated sample that represents OnTrackNY participants and family members in an urban area. Given that all study interviews were conducted online, this limited our ability to understand the experiences of those for whom technology was a major barrier to digital community participation during the pandemic. Additionally, our member check was not conducted with the same individuals who participated in the focus groups and interviews.

For individuals experiencing early psychosis, community participation is central to the recovery process. The COVID-19 pandemic created unprecedented barriers to community participation that impacted CSC participants. Yet, examples from the OnTrackNY CSC program demonstrated how participants, family members and providers developed innovative strategies to help young people continue remain involved in meaningful activities, cultivate a sense of agency, and engage with others. Within the new context for care delivery since the COVID-19 pandemic, it is important that CSC programs promote community participation in traditional community settings as well as reduce barriers by offering opportunities for digital community participation.
